# Risk factors for ill health: How do we specify what is ‘modifiable’?

**DOI:** 10.1371/journal.pgph.0002887

**Published:** 2024-03-04

**Authors:** Nisreen A. Alwan, Seb Stannard, Ann Berrington, Shantini Paranjothy, Rebecca B. Hoyle, Rhiannon K. Owen, Simon D. S. Fraser

**Affiliations:** 1 School of Primary Care, Population Sciences and Medical Education, Faculty of Medicine, University of Southampton, Southampton, United Kingdom; 2 University Hospital Southampton NHS Foundation Trust, Southampton, United Kingdom; 3 Department of Social Statistics and Demography, Faculty of Social Science, University of Southampton, Southampton, United Kingdom; 4 School of Medicine, Medical Sciences and Nutrition, University of Aberdeen, Aberdeen, United Kingdom; 5 School of Mathematical Sciences, University of Southampton, Southampton, United Kingdom; 6 Swansea University Medical School, Faculty of Medicine, Health and Life Sciences, Swansea University, Swansea, United Kingdom; PLOS: Public Library of Science, UNITED STATES

If you work in public health or epidemiology, you will be familiar with the term ‘modifiable risk factors’. Searching PubMed in December 2023 for titles that included the term ‘modifiable risk’ returned 1222 results. Expanding this search to include both titles and abstracts returned 13,958 results. The term often refers to health behaviours such as smoking, alcohol intake, exercise, and diet. However, there does not seem to be a specific definition of what is classed as ‘modifiable’ in the context of the risk of ill health. Although the term is also used to refer to factors indirectly affecting health, such as education or housing, current use tends to focus on individual behaviours, largely neglecting the role of systemic and structural determinants of disease and health inequities.

## What does a ‘modifiable risk factor’ mean?

The Cambridge Dictionary defines the term ‘modify’ as ‘to change something such as a plan, opinion, law or way of behaviour slightly, usually to improve it or make it more acceptable’ [1]. In epidemiology, the term ‘modifiable’ is often poorly defined, and frequently used without explaining how a factor is designated as such. In statistics it would usually be used to describe a variable, factor, or parameter that can be changed or manipulated in some way.

Classification of what constitutes a modifiable risk factor varies across the medical and social science literature. For example, some classify age, race, and chronic health conditions as ‘non-modifiable’ [2]. However, in certain contexts we can argue that age as a risk indicator for health can be modified [3] -for example, the age at which women become pregnant. In other contexts, age may be closely related to frailty but independent from it, and therefore may not be considered modifiable.

Race and/or ethnicity are social constructs which are closely related to other determinants of ill health such as poverty, isolation, and discrimination -mechanisms that produce poorer health outcomes and are potentially modifiable [4]. Race and ethnicity as non-modifiable factors are commonly used as proxy for underlying biological processes or as a genetic category: in doing so, the identification of structural causes of ill health may become obscured [5]. Even when considering genetics, with the emerging evidence around the role of epigenetics in shaping chronic disease risk, we can perhaps say that this is at least partially modifiable [6].

Some long-term conditions are arguably modifiable. For example, remission of type 2 diabetes may be achieved if people lose weight, hypertension can potentially be reversed with behavioural changes, and for some people kidney function is variable, with chronic kidney disease appearing to ’remit’ [7, 8].

## What counts as ‘modifiable’ depends on the context, but can we agree basic universal criteria for definition?

In some contexts, the purpose of classifying a risk factor as modifiable is a call for action at the individual or group level. In others, a wider definition of ‘modifiable’ may be warranted such as when considering aetiological associations with health outcomes, as well as accounting for moral, ethical, and human rights dimensions of causality.

The potential for modification could be considered at the individual, community, national, or global levels. This includes the social, economic, political, commercial, and environmental determinants of health. Clearly articulating what is meant by ’modifiable’ gives us the ability to develop common understanding, consensus, definition, and application of the term, while fully recognising that such consensus may very much be context- and purpose-dependent.

## Are there any general criteria to consider when specifying factors as ‘modifiable’ in the context of health research?

**Fig 1:**
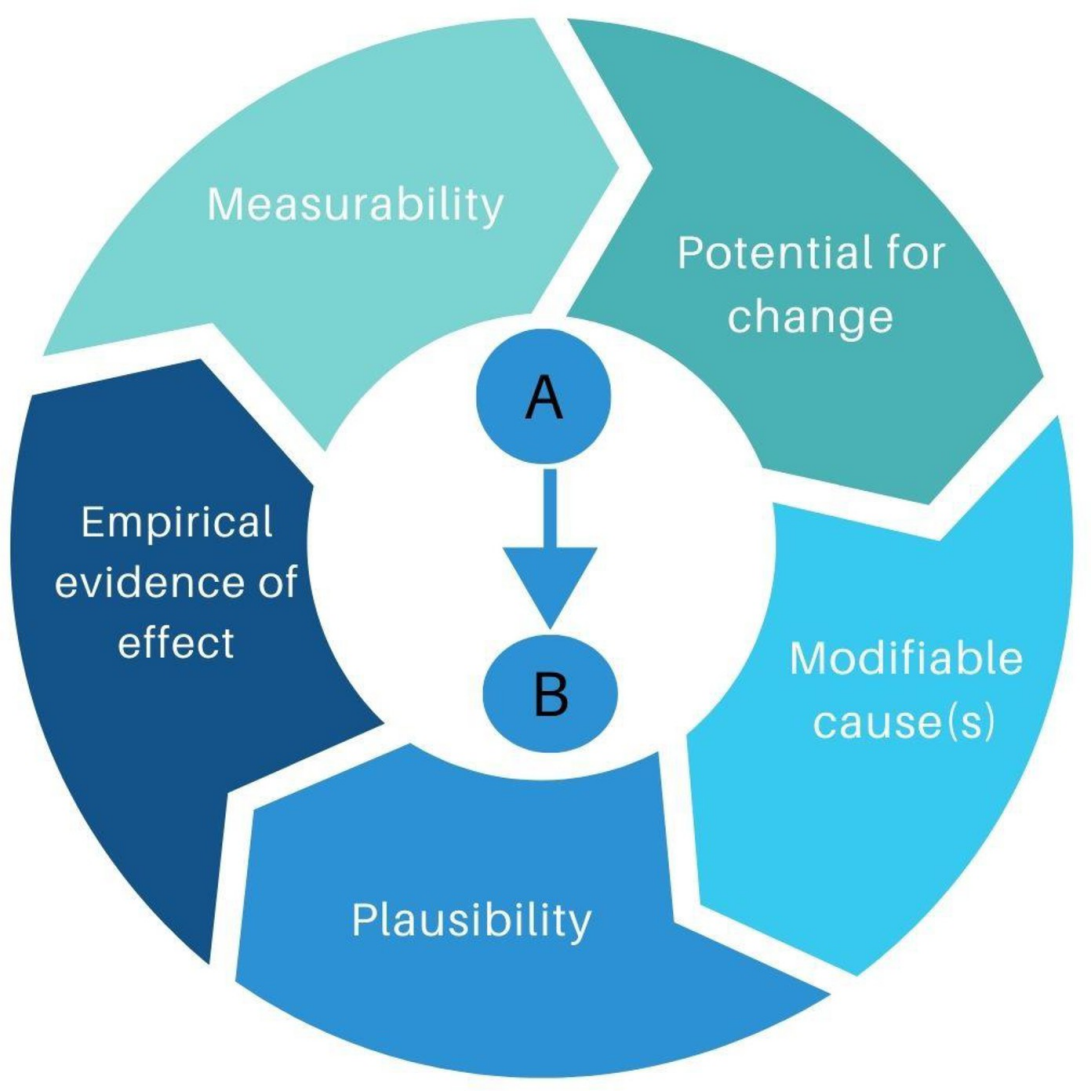
Criteria to consider before classifying health risk factors or determinants as ‘modifiable’.

The following questions can help us define what we are considering as modifiable risk factors of health and disease outcomes (Fig 1):

### 1. Is it measurable?

Can the factor under consideration be measured? Without the ability to measure, consideration for the rest of the criteria cannot be operationalised. For an individual-based exposure example, smoking can be measured by its presence or absence, dose, and type.

### 2. Is it potentially changeable?

Is there potential for change in the quantity and intensity of the modifiable factor at either individual or population level? For example, smoking is potentially changeable with direct intervention at the individual level (smoking cessation programmes), or population level (raising prices, taxation) [9]. It can also be potentially changeable with indirect intervention at the individual level such as interventions targeting psychological distress [10]. Another example of an indirect intervention at an individual level is rent assistance influencing behaviours such as substance abuse and problem drinking [11]. At a population level, curriculum changes to promote self-esteem and inclusive ethos at school may modify maternal age at conception which in turn may reduce adverse health outcomes of teenage pregnancy [12].

### 3. Are its causes modifiable in themselves?

In health research, we usually assess the association of an exposure to an outcome, accounting for confounding and mediation by other variables. In epidemiology, this is commonly determined by conceptualisation of causal diagrams or the use of directed acyclic graphs [13]. Factors that are ‘parents’ of the exposure of interest can be assessed for their potential modifiability too. For example, living in a deprived area is a cause of higher mortality in people with COVID19 [14]. Type of occupation and household income can be considered ‘parents’ to ‘living in a deprived area’, and they are themselves potentially modifiable.

### 4. Is it plausible as a cause?

Applying the Bradford-Hill criteria [15] for consideration of causation may be helpful in conceptualising whether factors are mechanistically plausible as causes or not. Using the COVID19 example above, certain types of frontline occupations such as healthcare, social care or teaching can plausibly lead to increased exposure to SARSCoV2, and this increased risk is plausibly mediated by living in a more deprived area. However, this may not fully explain the increased associated mortality among those infected and plausible explanations of confounding such as crowded housing, comorbidities, or inequities in healthcare access may act as competing causes.

### 5. Is there empirical evidence for its effect?

Empirical evidence of direct effect on health outcomes can strengthen the case for considering factors modifiable, such as evidence from a randomised controlled trial on the effect of a smoking cessation programme on cardiovascular disease risk. However, this is not essential if the other criteria are satisfied and change in outcome by removing or changing the dose of the exposure is demonstrated via an indirect route. The degree of change in outcome because of modifying a particular factor or the extent to which a factor operates individually as a cause as demonstrated by empirical evidence are issues for further deliberation. For example, how much of cardiovascular risk is eliminated with smoking cessation programmes as opposed to other modifiable factors.

## Conclusion

We conceptualised the above questions for application in our own empirical research. The next step is to establish consensus. In the absence of an agreed definition, transparent criteria to define what can be considered as modifiable determinants of health are desperately needed. An explicit consideration of what can be considered “modifiable” can facilitate more comprehensive public health planning and make the concept of modification more inclusive of the wider determinants of health. This is urgently needed if we want to tackle health inequities both on the global and within-country scales.

## References

[1] Cambridge Dictionary [Internet]. Modify. 2023 [Cited 2024 January 4]. Available from: https://dictionary.cambridge.org/dictionary/english/modify

[2] Midha S, Chawla S, Garg PK. Modifiable and non-modifiable risk factors for pancreatic cancer: A review. Cancer Lett. 2016;381(1): 269–277. doi: 10.1016/j.canlet.2016.07.022 27461582

[3] Bennetsen AKK, Faber MT, Nygaard M, Sundstrom K, Hansen BT, Munk C, et al. Factors associated with teenage pregnancy in the Scandinavian countries. Scand J Public Health. 2023;0(0). doi: 10.1177/14034948231172819 37165576

[4] Braveman P, Parker DT. Abandon “Race.” Focus on Racism. Front Public Health. 2021;9: 689462. 34557466 10.3389/fpubh.2021.689462 PMC8452910

[5] Michos ED, Ferdinand KC, Brewer LC, Bond RM, Wong ND. Response to the letter to the editor by Silverman-Lloyd et al. entitled: "Race is not a risk factor: Reframing discourse on racial health inequities in CVD prevention". Am J of Prev Cardiol. 2021;6: 100188. 34327507 10.1016/j.ajpc.2021.100188 PMC8315448

[6] González-Becerra K, Ramos-Lopez O, Barrón-Cabrera E, Riezu-Boj JI, Milagro FI, Martinez-Lopez E, et al. Fatty acids, epigenetic mechanisms and chronic diseases: a systematic review. Lipids Health Dis. 2019;18: 178. doi: 10.1186/s12944-019-1120-6 31615571 PMC6792183

[7] Lean ME, Leslie WS, Barnes AC, Brosnahan N, Thom G, McCombie L, et al. Primary care-led weight management for remission of type 2 diabetes (DiRECT): an open-label, cluster-randomised trial. Lancet. 2018; 10;391(10120): 541–551. doi: 10.1016/S0140-6736(17)33102-1 29221645

[8] Hirst JA, Taal MW, Fraser SD, Mena JMO, O’Callaghan CA, McManus RJ, et al. Change in glomerular filtration rate over time in the Oxford Renal Cohort Study: observational study. Br J Gen Pract. 2022; 31;72(717): e261–e268. doi: 10.3399/BJGP.2021.0477 34990394 PMC8869187

[9] Brown T, Platt S, Amos A. Equity impact of population-level interventions and policies to reduce smoking in adults: A systematic review. Drug Alcohol Depend. 2014;138: 7–16. doi: 10.1016/j.drugalcdep.2014.03.001 24674707

[10] Siegel A, Korbman M, Erblich J. Direct and Indirect Effects of Psychological Distress on Stress-Induced Smoking. J Stud Alcohol Drugs. 2017;78(6): 930–937. doi: 10.15288/jsad.2017.78.930 29087829 PMC5668998

[11] Acevedo-Garcia D, Osypuk TL, Werbel RE, Meara ER, Cutler DM, Berkman LF. Does Housing Mobility Policy Improve Health? Hous Policy Debate. 2004;15(1), 49–98.

[12] Fletcher A, Harden A, Brunton G, Oakley A, Bonell C. Interventions addressing the social determinants of teenage pregnancy. Health Educ. 2008;108(1): 29–39.

[13] Suttorp MM, Siegerink B, Jager KJ, Zoccali C, Dekker FW. Graphical presentation of confounding in directed acyclic graphs. Nephrol Dial Transpl. 2015;30(9): 1418–1423. doi: 10.1093/ndt/gfu325 25324358

[14] McGowan VJ, Bambra C. COVID-19 mortality and deprivation: pandemic, syndemic, and endemic health inequalities. Lancet Public Health. 2022;7(11): e966–e975. doi: 10.1016/S2468-2667(22)00223-7 36334610 PMC9629845

[15] Shimonovich M, Pearce A, Thomson H, Keyes K, Katikireddi SV. Assessing causality in epidemiology: revisiting Bradford Hill to incorporate developments in causal thinking. Eur J Epidemiol. 2021;36: 873–887. doi: 10.1007/s10654-020-00703-7 33324996 PMC8206235

